# Vascular Reconstructions in Living Unrelated Kidney Transplant Using Donor Ovarian Vein and Recipient Inferior Epigastric Artery with Simultaneous Enucleation of a Complex Cyst

**DOI:** 10.1155/2019/3272080

**Published:** 2019-03-13

**Authors:** Giuseppe Serena, Javier Gonzalez, Giselle Guerra, Mohamed Ammar Al Nuss, Maykel Valdes, Gaetano Ciancio

**Affiliations:** ^1^Department of Surgery, University of Miami Miller School of Medicine, Jackson Memorial Hospital, Miami, FL, USA; ^2^Miami Transplant Institute, University of Miami Miller School of Medicine, Jackson Memorial Hospital, Miami, FL, USA; ^3^Servicio de Urologia, Hospital General Universitario Gregorio Marañón, Madrid, Spain; ^4^Department of Medicine, Division of Nephrology, Miami Transplant Institute, University of Miami Miller School of Medicine, Jackson Memorial Hospital, Miami, FL, USA; ^5^Department of Urology, University of Miami Miller School of Medicine, Jackson Memorial Hospital, Miami, FL, USA

## Abstract

Increasing the organ donor pool and solving the recipient demands continue to be one of the challenges in transplantation. We report our experience in transplanting a living donor kidney requiring complex vascular reconstructions and an enucleation of complex cyst. A 57-year-old male patient underwent a living unrelated kidney transplant. The living donor kidney was procured through a laparoscopic hand-assisted right donor nephrectomy. After vascular stapling, the kidney had a short upper pole arterial branch, a short renal vein (3 mm), and a complex upper pole cyst. The renal vein was elongated using the donor ovarian vein and the short upper pole artery was extended using the recipient inferior epigastric artery and anastomosed to the main renal artery. The renal allograft vessels were anastomosed end-to-side to the external iliac vessels. The complex cyst was removed performing an enucleation with a rim of normal tissue and reconstruction of the calyceal system. This case represents three different surgical reconstructions in order to make the organ available for transplantation. In some circumstances, complex vascular reconstruction of living donor kidney with removal of complex cyst represents a strategy to expand the donor pool.

## 1. Introduction

Kidney transplantation offers a higher life expectancy and a better quality of life compared to dialysis [1]. Renal transplant represents the best treatment for patients with end stage renal disease (ESRD). However, the median waiting time for adult kidney transplant has increased over time. From 2005 to 2015, candidates waiting more than 5 years increased from 11.4% to 15.7% [2]. Using kidneys with vascular and parenchymal issues is part of a debatable scenario and leaves room for discussion to help address donor expansion.

We describe a case of a living kidney donor that after vascular stapling had a short donor renal vein (3 mm), a short donor upper pole arterial branch, and a complex renal cyst. The donor renal vein was reconstructed using the donor ovarian vein; the recipient inferior epigastric artery (IEA) was used for the short upper pole artery; and enucleation with a rim of normal tissue was performed to remove the complex renal cyst.

## 2. Case Presentation

The patient was a 57-year-old male, with ESRD secondary to diabetes and hypertension, on hemodialysis for 20 months. He was evaluated to undergo living unrelated donor kidney transplant. The donor was a 54-year-old female with unremarkable past medical history. She was medically and surgically cleared after full assessment. Preoperative computed tomography (CT) angiography for the donor revealed a small complex cyst (Bosniak IIF), a short right renal vein (2 cm), and a right renal artery with an early bifurcation of an upper pole artery. Surgical approach deemed best by the living donor selection committee was to remove the right kidney with the complex cyst via laparoscopic approach.

### 2.1. Description of the Procedure

The risks of surgery and potential complications were explained to both donor and recipient. Written informed consent was obtained prior to surgery from both patients.

A standard hand-assisted laparoscopic living donor nephrectomy (LLDN) was performed to retrieve the right kidney and the right ovarian vein for reconstruction. The length of the donor renal vein was reduced by 10-15 mm due to the standard use of vascular stapler device to control the renal vein stump.

**Figure 1:**
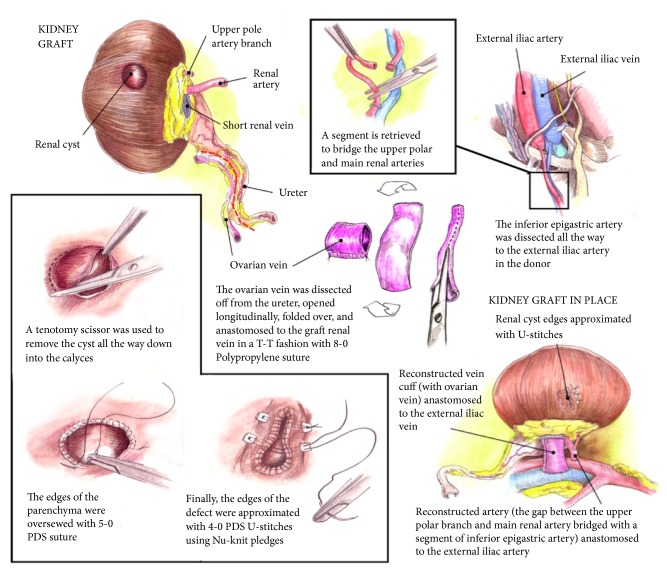
Schematic drawing describes the three surgical anomalies of the right donor kidney.

Status after procurement upon visualization in the back table is that the length of the donor renal vein was 3 mm. In order to elongate the vein, the donor ovarian vein was dissected and used for reconstruction. It was opened longitudinally, folded over, and anastomosed to the donor renal vein using end-to-end anastomosis with an 8-0 Prolene (Figure 1).

The donor kidney had a renal artery with early bifurcation of an upper pole artery. After mobilization of the inferior vena cava as much as possible, the endovascular stapler was placed after the bifurcation from the renal artery and the length of the vessel was reduced by 10-15 mm. At the back table the donor kidney presented two individual arteries: the main renal artery and the short upper pole arterial branch. Consequently, the recipient IEA was used as an extension graft to perform an end-to-side anastomosis between the upper pole arterial branch and the main renal artery using 8-0 Prolene (Figure 1).

The complex cyst was dissected all the way down into the calyces and substantial margin of healthy parenchyma was removed. Distal margin was sent to pathology to rule out malignancy. The calyces and the renal parenchyma were oversewed with 5-0 PDS and the edges of the defect with U-stitch of 4-0 PDS using pledget (Figure 1).

The extended renal vein and the main renal artery were anastomosed end-to-side to the external iliac vein (EIV) and artery (EIA) respectively, using 6-0 Prolene (Figure 1).

We performed an extravesical ureteroneocystostomy and a Jackson Pratt drain was placed. The warm ischemia time was 33 minutes. Postoperative course was uncomplicated and the patient has maintained adequate renal function with stable serum creatinine (1.32 mg/dl) at 5-month follow-up. The final pathology report and the intraoperative frozen section of the margin were negative for malignancy.

## 3. Discussion

The left kidney has been considered the preferred one for living donor kidney transplant. However, the presence of a cyst in the right donor kidney or complex vascular anomalies of left donor kidney are indications for using the right kidney. According to Abrahams et al., the presence of a cyst in the right kidney is an absolute indication for selecting the right side for live donation [3]. Using the right kidney for living donor kidney transplant is considered more challenging due to the shorter length of the right renal vein and the increased risk of allograft thrombosis. Furthermore, the modern hand-assisted LLDN increased loss of the length of the vessels due to the vascular stapling [3].

Donor kidney with short right renal vein is associated with possible anastomotic complications such as excessive tension or acute angulation of renal vein anastomosis. Both of them increase postoperative thrombotic events in the donor renal vein [4]. Renal vein extension (RVE) is an option to improve the success rate of the short right vein anastomosis reducing operative complications of living donor kidney transplants (LDKT). Lin et al. described their experience using donor gonadal vein to elongate the right renal vein. Donor gonadal vein was previously extracted through the hand-assisted LLDN and used for vascular reconstruction to facilitate graft implantation in LDKT [5].

In our case we described how the vascular stapling shortened the upper pole arterial branch. Consequently we used the IEA as extension graft to perform an end to side anastomosis between a short upper pole arterial branch and the main renal artery. Short polar arteries have been an issue described overtime, mostly in kidney with multiple arteries. El-Sherbiny et al. reported the importance to reconstruct polar arteries in order to maintain an adequate perfusion of upper/lower pole of the kidney or the ureter itself [6]. To our knowledge, use of the IEA for bench reconstruction of upper pole artery of kidneys is the best vascular reconstruction technique [7].

Lastly and maybe most importantly is the discussion regarding the management of the complex cyst present in the donor kidney. Incidence of renal cyst in 50-year-old patients is between 17% and 39% [8]. Kidney Disease Improving Global Outcomes (KDIGO) reported that careful assessment of complex cystic masses should be done with preoperative CT scan and Bosniak system for radiological classification. Both Bosniak classification and proper CT imaging improve the diagnostic accuracy and are useful tools for surgical decision-making. Most importantly KDIGO clinical guideline 2017 states that Bosniak IIF or higher cyst should not be left in the donor kidney [9]. The importance of removing the complex cyst from the right donor kidney is due to the increased risk of developing malignancy. Smith and al. described their experience with 16 patients with Bosniak IIF cystic renal lesions. They surgically excised the complex cysts and reported that the malignancy rate in their patients was 25% [10].

In the scenario in which the donor kidney presents a complex cyst, one of the options for the transplant surgeon is conservative surgery, such as enucleation or partial nephrectomy. After radiological evaluation, enucleation can be used for small lesions in polar areas while still maintaining adequate renal function [11].

## 4. Conclusion

To our knowledge, this is the first such case to be reported in literature of living donor kidney with these three surgical challenges: short main renal vein, short upper pole renal artery branch, and a complex renal cyst. The case required a complex vascular reconstruction and an enucleation. Donor gonadal vein and recipient IEA were used as extended grafts and the complex renal cyst suspicious of malignancy was removed with an enucleation. However, all this effort was done with the intention of making the living donor kidney transplantable. The recipient had a successful outcome with a normal renal function and no postoperative complications.

In conclusion, we presented our experience in managing a complex surgical case in a living donor kidney.
